# Prognostic value of cancer antigen -125 for lung adenocarcinoma patients with brain metastasis: A random survival forest prognostic model

**DOI:** 10.1038/s41598-018-23946-7

**Published:** 2018-04-04

**Authors:** Hao Wang, Liuhai Shen, Jianhua Geng, Yitian Wu, Huan Xiao, Fan Zhang, Hongwei Si

**Affiliations:** 1 Department of Radiotherapy, The First Affiliated Hospital of Anhui Medical University, Hefei, Anhui Province 230022 China; 2 Department of Nuclear Medicine, The First Affiliated Hospital of Anhui Medical University, Hefei, Anhui Province 230022 China; 3 Department of Nuclear Medicine, National Cancer Center/ Cancer Hospital, Chinese Academy of Medical Sciences and Peking Union Medical College, Beijing, 100021 China

## Abstract

Using random survival forest, this study was intended to evaluate the prognostic value of serum markers for lung adenocarcinoma patients with brain metastasis (BM), and tried to integrate them into a prognostic model. During 2010 to 2015, the patients were retrieved from two medical centers. Besides the Cox proportional hazards regression, the random survival forest (RSF) were also used to develop prognostic model from the group A (n = 142). In RSF of the group A, the factors, whose minimal depth were greater than the depth threshold or had a negative variable importance (VIMP), were firstly excluded. Subsequently, C-index and Akaike information criterion (AIC) were used to guide us finding models with higher prognostic ability and lower overfitting possibility. These RSF models, together with the Cox, modified-RPA and lung-GPA index were validated and compared, especially in the group B (CAMS, n = 53). Our data indicated that the KSE125 model (KPS, smoking, EGFR-20 (exon 18, 19 and 21) and Ca125) was the best in survival prediction, and performed well in internal and external validation. In conclusions, for lung adenocarcinoma patients with brain metastasis, a validated prognostic nomogram (KPS, smoking, EGFR-20 and Ca125) can more accurately predict 1-year and 2-year survival of the patients.

## Introduction

Brain metastases (BM) from cancers were conventionally treated as a single disease entity^1^, and some pre-treatment prognostic factors had been investigated. Using the Radiation Therapy Oncology Group (RTOG) databases, Gaspar *et al*.^2^ proposed the recursive partitioning analysis (RPA) classes which were modified in 2012 (modified RPA)^3^. Based on the data from RTOG 9508, Sperduto *et al*.^4^ developed the graded prognostic assessment (GPA) index. Because BM exhibits obviously variability in clinical response and overall survival (OS)^1^, the GPA index was revised as the Diagnosis-Specific GPA (DS-GPA)^5^. These studies identified the prognostic factors of Karnofsky performance status (KPS), age, primary tumor status, extracranial metastases, and number of BM.

Lung adenocarcinoma is one of the diseases that frequently develop BM, and some disease-specific factors have been studied, such as, serum markers, epidermal growth factor receptor status (EGFR), tyrosine kinase inhibitor (TKI) therapy, and so on^6^. Recently, Sperduto *et al*.^7^ developed the Lung-molGPA, which included two new factors (EGFR and ALK alterations), but not serum marker. Therefore, this study was designed to investigate the prognostic value of some serum markers for the patients.

In developing prognostic models, variable selection is in such a dilemma. When more factors are integrated, the model from the training data set can be fitted more accurately (high overfitting possibility); however, it might performed badly in most other sets (low generalization ability)^8^. In other words, the overfitted model cannot accurately estimate the prognosis of other patients. Typically, as illustrated by the cited studies, prognostic factors for survival data are selected by the multivariate Cox proportional hazards regression with the criterion of those significantly against OS. Although the regression is popularly used, it suffers from high variance and poor performance, especially under the conditions involving multiple factors or nonlinear effects^9^. Random survival forest (RSF) is considered as a more accurate method for right-censored survival data. Based on bootstrap data and the majority votes of the individual decision trees, RSF can construct multiple decision trees to predict the outcome^10^, and models non-linear effects and complex interactions among factors^11^.

Above all, using random survival forest, this study was intended to evaluate the prognostic value of some serum markers for lung adenocarcinoma patients with brain metastasis, and tried to integrate them into a prognostic model.

## Materials and Methods

### Study Population

During 2010 to 2015, the patients with a history of lung adenocarcinoma were retrospectively reviewed from the cases at the First Affiliated Hospital of Anhui Medical University (AMU) and Cancer Hospital Chinese Academy of Medical Sciences (CAMS). The inclusion criteria were: pathologically verified lung adenocarcinoma (International Association for the Study of Lung Cancer, IASLC, eighth edition^12^), historically or newly diagnosed BM, and accepted the EGFR gene mutation detection and the laboratory examinations of CA125 (cancer antigen 125), Cy211 (cytokeratins -19 fragments), CA199 (cancer antigen 199), NSE (neuron specific enolase), CEA (carcinoembryonic antigen), SCC (squamous cell carcinoma antigen), and ProGRP (progastrin-releasing peptide). At the diagnosis of BM, besides the factors in the lung-GPA and the modified-RPA model, smoking which defined as more than 40 packs per year was also retrieved. From medical records or by telephone, the patients were followed to the end of November 2016. OS was the day BM diagnosed to death for any reason. Considering the sample size from the two centers, the patients from the AMU (group A) were used to train the prognostic model, which was externally validated by the data from the CAMS (group B). Additionally, to make sure the robustness of the RSF method, the group A was resampled and analyzed in the “Supplementary Data” part. Using SPSS software package, the recruited patients were randomly divided into the group SA (n = 115) and SB (n = 27), which was used to train and validate RSF models, respectively. The protocol was approved by the ethics committee at the AMU and the CAMS.

Qiagen formalin fixed paraffin embedded (FFPE) DNA extraction kit was used to extract genomic DNA. Exons 18, 19, 20 and 21 of the extracted DNA were amplified by polymerase chain reaction (PCR) technique, and were analyzed by direct Sanger sequencing^13^. Because NSCLC patients with EGFR exon 20 insertion were not well respond to gefitinib or erlotinib as those with other mutations^14^, EGFR mutation status was analyzed under two classifications, ie EGFR (exon 19–21) and EGFR-20 (exon 18, 19 and 21).

### Variable Selection

RSF classifier can select prognostic factors by two indicators: minimal depth and variable importance (VIMP). Minimal depth is the node number from the root node to the parent node of the factor located. The smaller the minimal depth of a factor is, the more ability it has on prediction. Furthermore, the mean number of minimal depth distribution of factors is the threshold value for variable selection, and can be used to decide whether a minimal depth of a factor is small enough as a powerful one^15^. VIMP is a comparable measurement of a factor in predicting the response or causal effect^16^, and is decreased with the increase in prediction error if the factor is randomized^10^. Zero or negative VIMP was not predictive^17^, which could be discarded in further analysis. Above all, minimal depth threshold and VIMP could help us to exclude some factors with low prognostic ability.

However, using all remaining factors to develop a prognostic model might result in overfitting, and may describe random error instead of the underlying relationship. Akaike information criterion (AIC) measures the relative quality of statistical models for a given dataset, and a lower value indicates higher quality and lower overfitting possibility. Therefore, it could be used to step-by-step select variables for developing models^18^. Based on the variable selection method, besides AIC, we also introduced another indicator of concordance index (C-index) to guide us developing potentially eligible models with both lower overfitting possibility and higher prognostic ability. C-index is similar to the area under a receiver operating characteristic (ROC) curve, and a higher percentage indicates higher prognostic ability^19^. Above all, a lower AIC and higher C-index of a model had, and the more explanatory and informative of the model was.

The nomogram of the best model was plotted by the Regression Modeling Strategies package (rms). A calibration plot (bootstrap = 1000) of its predictions were plotted against the observed probabilities. An accurate prognostic nomogram has a plot where the observed and predicted probabilities for given groups fall along the 45-degree line^20^.

### Internal and External Validation of Prognostic Nomogram

Internal validation was used to select the best from the potentially eligible RSF models, which were also compared with current models (modified RPA and Lung-GPA). Besides C-index (discriminatory ability) and AIC (overfitting possibility), these models were also compared by out-of-bag (OOB) error to estimate the generalization error. Among randomly growing RSF trees, about one-third of the cases are not used for training (OOB data), and can be used to unbiasedly estimate the classification error when trees are added to the forest^21^. Therefore, a lower OOB error indicates a better RSF model.

Additionally, a 10 fold cross-validation was also used to internally validate the performance of these models^22^. The validation method randomly divided the original dataset into10 equal sized subsets, and the model is repeatedly trained and validated 10 times. At each time, 9 subsets are pooled to train the model, and then the model is validated in the retained subset. The average error across 10 rounds is the indicator (integrated Brier score, IBS) for generalizing the model in an independent dataset^23^, and a lower IBS indicates a better model.

All data was analyzed with the R project (version 3.3.1). The important software packages for the R project included pec, rms, and randomForestSRC. A two sides of p < 0.05 was considered as the significant level.

### Ethics approval and informed consent

Our protocol was approved by the ethics committee of the First Affiliated Hospital of Anhui Medical University and the National Cancer Center/Cancer Hospital, Chinese Academy of Medical Sciences and Peking Union Medical College. The study was conducted in accordance with the relevant guidelines and regulations. Informed consent was obtained from all participants according to the institutional guidelines.

### Data availability statement

The datasets used and/or analyzed during the current study are available from the corresponding author on reasonable request.

## Results

### Patient Characteristics

Patient characteristics of the group A and B are listed in Table 1, and those of the group SA and SB are in Table S1 (Supplementary Data). The median age of the group A (AMU) and B (CAMS) were 57 y (n = 142, 28 y to 79 y) and 57 y (n = 53, 24 y to 76 y), respectively. In the group A and B, 98/142 and 6/53 patients already had BM at the time of lung cancer diagnosed (*x*^2^ = 51.615, P = 0.000), and others (n = 44 and 47) developed BM during 0.0–43.1 months (median 5.3 months) and 0.0–87.0 months (median 10.9 months), respectively.

**Table 1:** Patient characteristics and univariate Cox regression.

Characteristic		Group A			Group B		
		n (%)	1-year OS (%)	Median (moths)	n (%)	1-year OS (%)	Median (moths)
Gender	Male	70 (49.3)	43.3	9.6	25 (47.2%)	59.0	15.8
	Female	72 (50.7)	49.9	12.0	28 (53.8%)	73.3	20.7
Age (year)	<50 y	36 (25.4)	50.6	15.2	13 (24.5%)	60.6	15.3
	50–59 y	49 (34.5)	48.8	11.7	16 (30.2%)	73.1	20.7
	≥60 y	57 (40.1)	42.4	9.0	24 (45.3%)	66.2	26.0
Smoking	No	87 (61.3)	48.8	12.0	34 (64.2%)	75.0	24.0
	Yes	55 (38.7)	42.8	9.0	19 (35.8%)	51.1	15.8
CEA	Normal	68 (47.9)	48.7	11.4	24 (45.3%)	60.6	15.0
	Abnormal	74 (52.1)	44.7	10.1	29 (54.7%)	71.5	26.0
CA199	Normal	103 (72.5)	46.0	10.2	46 (86.8%)	66.8	20.0
	Abnormal	39 (27.5)	47.6	12.0	7 (13.2%)	68.6	20.0
SCC	Normal	137 (96.5)	47.3	11.4	49 (92.5%)	64.4	17.0
	Abnormal	5 (3.5)	26.7	12.0	4 (7.5%)	NA	24.0
NSE	Normal	135 (95.1)	46.7	11.4	51 (96.2%)	67.5	20.0
	Abnormal	7 (4.9)	42.9	6.9	2 (3.8%)	50.0	1.8
Primary	Controlled	19 (13.4)	62.0	15.2	34 (64.2%)	67.8	17.0
tumor	Uncontrolled	123 (86.6)	44.7	10.1	19 (35.8%)	64.8	24.0
Extracranial	Yes	110 (77.5)	44.6	9.7	9 (17.0%)	77.8	27.0
metastases	No	32 (22.5)	53.8	12.5	44 (83.0%)	64.0	20.0
NoBM	1	51 (35.9)	55.0	13.3	18 (34.0%)	81.0	NA
	2–3	41 (28.9)	39.9	9.7	19 (35.8%)	51.6	26.0
	>3	50 (35.2)	43.5	9.3	16 (30.2%)	68.2	17.0
ProGRP	Normal	141 (99.3)	47.0	11.4	51 (96.2%)	65.4	20.0
	Abnormal	1 (0.7)	0.0	6.9	2 (3.8%)	50.0	17.0
Treatment	No treatment	17 (12.0)	17.6	4.4	1 (1.9%)	NA	NA
	Chemotherapy	42 (29.6)	50.5	15.5	26 (49.0%)	49.7	11.3
	Radiotherapy	28 (19.7)	59.2	14.4	1 (1.9%)	NA	NA
	Combined	39 (27.5)	48.2	11.4	24 (45.3%)	83.1	26.0
	TKI alone	16 (11.3)	53.0	10.1	1 (1.9%)	NA	NA
EGFR	wt	73 (51.4)	42.3*	9.7	26 (49.1%)	51.0	15.8*
	mut	69 (48.6)	51.1	12.5	27 (50.9%)	80.7	30.0
EGFR-20	wt	76 (53.5)	42.0*	9.6	26 (49.1%)	51.0	15.8*
	mut	66 (46.5)	51.8	12.5	27 (50.9%)	80.7	30.0
Cy211	Normal	45 (31.7)	62.3*	15.5	34 (64.2%)	70.9	17.0
	Abnormal	97 (68.3)	39.8	9.2	19 (35.8%)	60.5	24.0
CA125	Normal	74 (52.1)	57.6**	14.8	37 (69.8%)	61.8	15.8
	Abnormal	68 (47.9)	34.4	8.6	16 (30.2%)	79.1	24.0
TKI therapy	No	83 (58.5)	38.1*	8.6	28 (52.8%)	51.3	15.8
	in wt pt	12 (8.5)	65.6	15.2	6 (11.3%)	50.0	7.0
	In mut pt	47 (33.1)	57.3	16.3	19 (35.8%)	89.5	30.0
BM	Already	98 (69.0)	48.7*	11.8	6 (11.3%)	NA*	NA
	Developed	44 (31.0)	42.7	8.0	47 (88.7%)	62.0	15.8
KPS	<70	40 (28.2)	7.8**	3.2	12 (22.6%)	0.00**	4.0
	70–80	86 (60.6)	53.8	13.3	31 (58.5%)	91.5	26.0
	90–100	16 (11.3)	93.8	33.4	10 (18.9%)	70.0	24.0
modified-RPA	Classes I	1 (0.7)	NA**	NA	13 (24.5%)	91.7**	20.0
	Classes II	101 (71.1)	60.3	15.2	29 (54.7%)	79.7	26.0
	Classes III	40 (28.2)	7.8	3.2	11 (20.8%)	0.0	4.0
lung-GPA	0–1	52 (36.6)	27.4**	6.0	6 (11.3%)	66.7	17.0
	1.5–2.5	77 (54.2)	52.9	12.5	37 (69.8%)	57.5	15.0
	3	9 (6.3)	77.8	16.4	6 (11.3%)	100.0	NA
	>3.5	4 (2.8)	66.7	33.4	4 (7.5%)	100.0	20.0

The significance between subsets in univariate Cox regression (*P < 0.05; **P < 0.01); NA: Not arrival.

Some patient characteristics were significantly different between the groups, such as, the serum markers of CA199 (*x*^2^ = 4.352, P = 0.037), Cy211 (*x*^2^ = 16.875, P = 0.000) and CA125 (*x*^2^ = 4.930, P = 0.026), and the prognostic models of modified -RPA (*x*^2^ = 32.882, P = 0.000) and lung-GPA (*x*^2^ = 13.259, P = 0.004). Additionally, compared to the group B, more patients in the group A already had BM (98/142 vs. 6/53, *x*^2^ = 51.615, P = 0.000), and presented extracranial metastases (104/142 vs. 9/53, *x*^2^ = 50.128, P = 0.000).

Furthermore, in the group A, some characteristics were not balanced between already and developed BM patients, for example, KPS scores, primary tumor status, extracranial metastases, and modified-RPA. Generally, compared to the patients already BM, the developed BM patients tended to have poorer performance status and present extracranial metastases. Although, modified-RPA (*x*^2^ = 14.886, P < 0.01) was significantly different between the already and developed BM patients, lung-GPA (*x*^2^ = 3.443, P > 0.05) was not.

### Treatment

Among the patients had received at least one cycle of chemotherapy (n = 155), the regimens of 112 patients could be followed. The most (n = 74) were treated with cisplatin (nedaplatin or lobaplatin) and one of paclitaxel (n = 2), docetaxel (n = 14), gemcitabine (n = 14), pemetrexed (n = 31), or vinorelbine (n = 13), and others (n = 38) were treated with carboplatin and one of paclitaxel (n = 4), docetaxel (n = 5), gemcitabine (n = 12), pemetrexed (n = 13), or vinorelbine (n = 4).

Among all patients (n = 195), 99 individuals received the whole brain radiotherapy by the Varian 6-MV linear accelerators. Additionally, radiotherapy was given to 49 patients for primary tumor or/and metastases (n = 6 for radiotherapy alone and n = 42 for combined treatment). Among those with available information (n = 33), 12 and 21 patients received conventional radiotherapy and intensity modulated radiation therapy (IMRT), and their median doses were 48 Gy (20–66 Gy) and 60 Gy (20–70 Gy), respectively.

**Figure 1:**
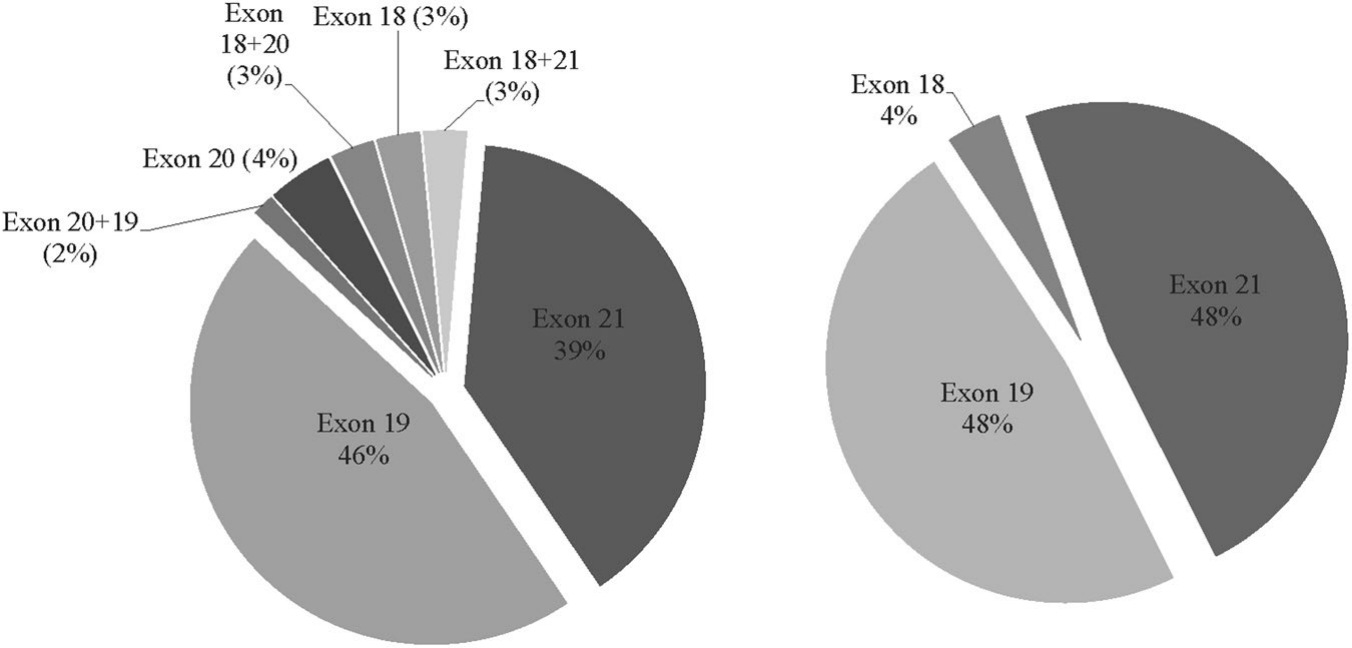
Exon mutations of EGFR gene. Left and right are for the group A and B, respectively.

In the group A and B, EGFR mutation (Exon 18–21) was detected in 69/142 and 27/53 patients (Fig. 1), who received TKI therapy in 49/69 and 19/27 patients, respectively. Among those accepted TKI therapy, the most (59/61 and 20/25) were after BM both in the group A and B, respectively. Besides those received TKI therapy alone (22/142 and 1/53 in the group A and B), 32, 1 and 6 patients of the group A also accepted chemotherapy, radiotherapy and combined treatment, and in 8, 0, and 16 patients of the group B, respectively.

Treatment modalities of the patients are also presented in Table 1. Between the group A and B, treatment (*x*^2^ = 26.915, P = 0.000) and primary tumor control (19/142 vs. 13/53, *x*^2^ = 50.264, P = 0.000) were significantly different, and the difference of treatment mainly resulted from that less patients in the group A received the combined treatment (19/142 vs. 24/53). In the group A, the treatment modalities were not significant against OS (*x*^2^ = 9.205, p = 0.056); however, TKI therapy (Table 1) was significant against OS (*x*^2^ = 7.287, p = 0.026), which resulted from the significance between TKI therapy and no TKI therapy (*x*^2^ = 6.992, p = 0.008).

### Survival and Cox Model

By the end of November 2016, in the group A and B, 92/142 and 27/53 patients died within 0.5 to 33.4 months (median 6.6 months) and 0.6 to 30 months (median 10 months). After excluding those without any treatment, the median OS was 9.0 (0.5–54.8) months and 13.3 (0.6–42.0) months, respectively.

The Kaplan-Meier analysis indicated that groups were significant factor against OS (*x*^2^ = 6.474, P = 0.011). Other significant factors were already or developed BM, TKI therapy, EGFR (or EGFR-20), Cy211, Ca125 and KPS in the group A, and were already or developed BM and KPS in the group B (Table 1).

**Figure 2:**
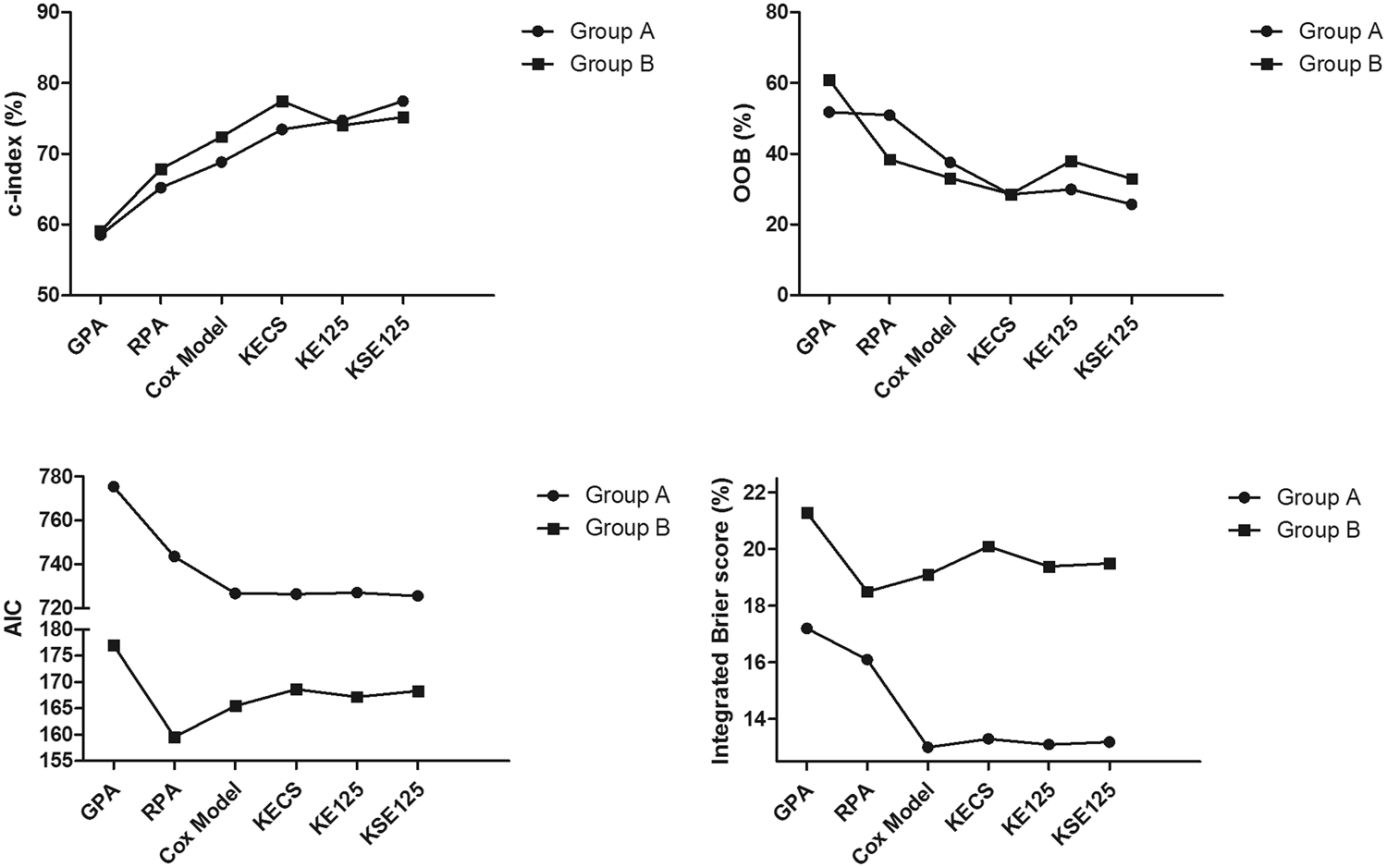
Comparison of prognostic models for lung adenocarcinoma patients with brain metastasis in the two groups.

In the multivariate Cox regression of the group A, EGFR (OR: 0.397, 95% CI: 0.397–0.942) and KPS (OR: 4.444, 95% CI: 2.940–6.717) were significant factors, which were confirmed by Table S2 (Supplementary Data). However, Fig. 2 indicates that C-index (or area under ROC) for the Cox model, modified-RPA, and lung-GPA are not so high enough. Therefore, we tried to build a powerful model by step-by-step RSF.

### RSF Models

**Figure 3:**
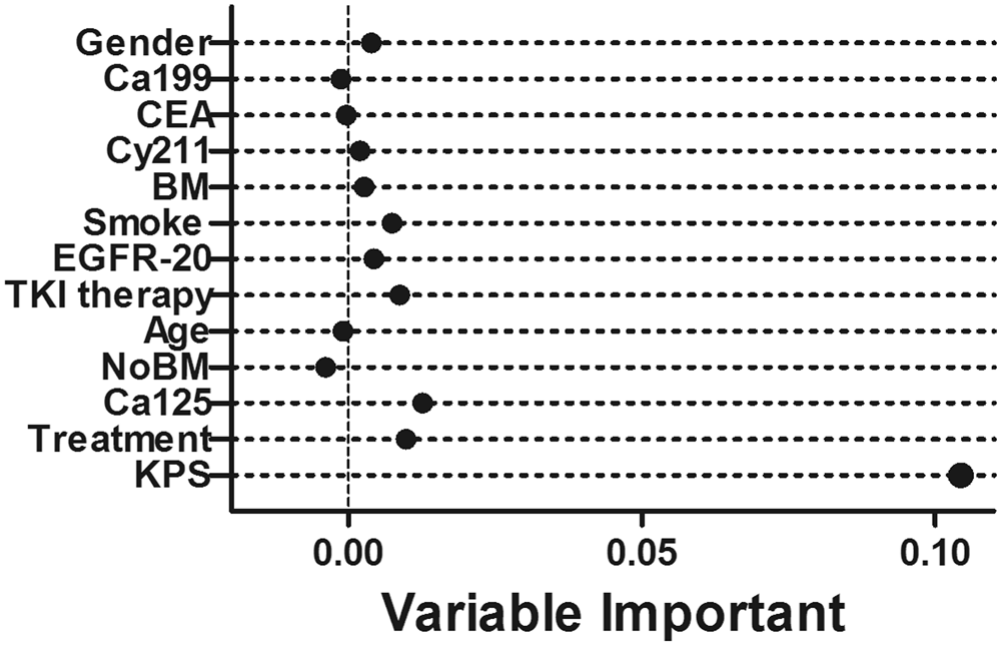
Scatter plot of VIMP against minimal depth. The variables on y-axis are sorted by minimal depth, and the smaller is closer to the origin. Additionally, only variables with a minimal depth lower than the threshold (4.6023) are plotted.

The data from the group A were used to train RSF models (results of group SA were in the Supplementary Data). Because the VIMP of EGFR-20 (exon 18, 19 and 21) was obviously higher than EGFR’s (exon 18–21), it was used in constructing RSF models (VIMP: 0.0073 vs. 0.0032). Minimal depth and VIMP of variables are plotted in Fig. 3. Among those variables below the minimal depth threshold (4.6023), nine variables had positive VIMP scores, and were qualified for further analysis (already or developed BM, KPS, Treatment, Ca125, TKI therapy, Cy211, EGFR-20, smoking and gender).

**Figure 4:**
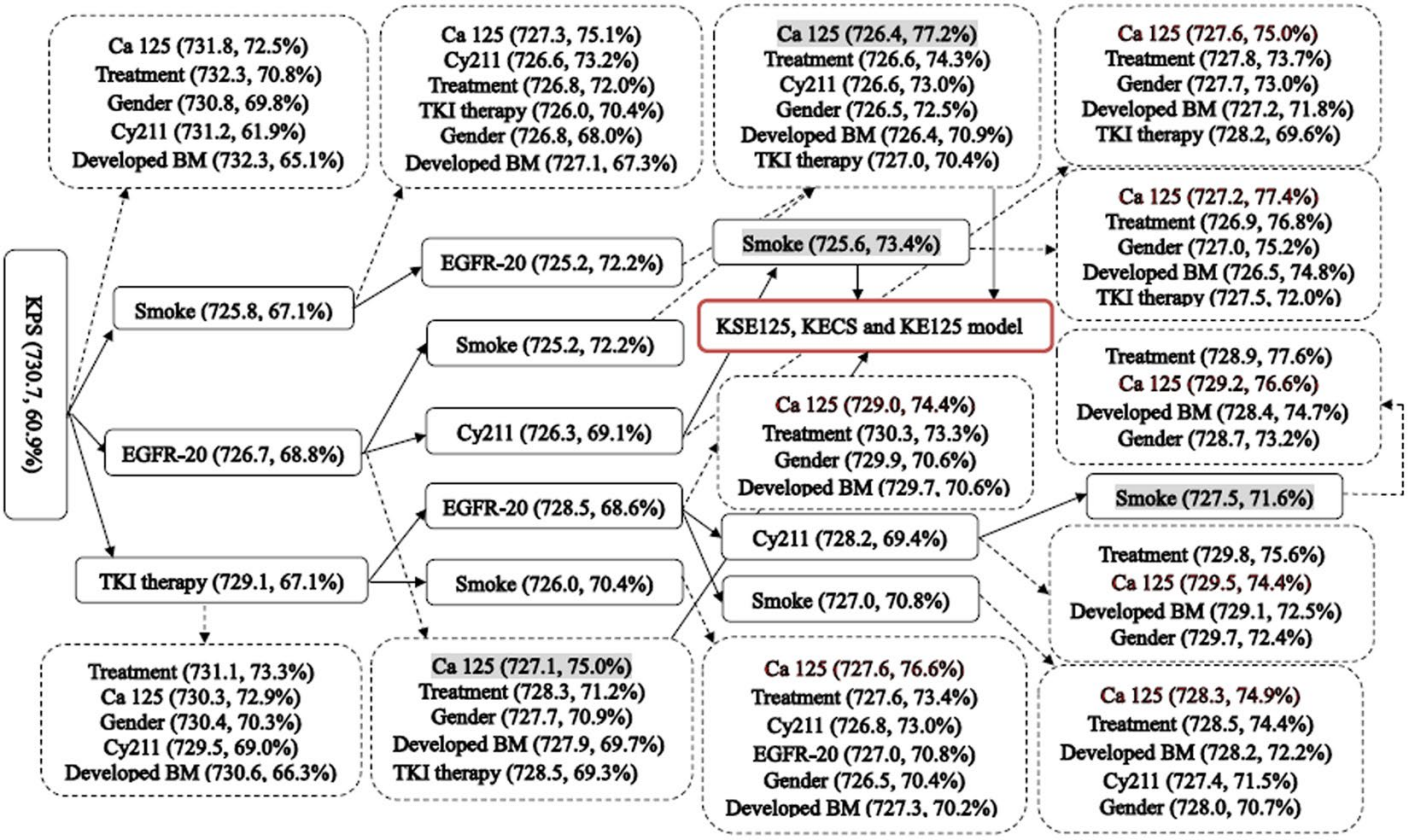
Variable selected by AIC and C-index. Broken lines indicate the ruled out variables. The last variable of eligible models are marked in grey background (n = 4). KTSCS model (KPS, TKI therapy, EGFR-20, Cy211 and smoking) is not selected for relative lower C-index (71.6%). Finally, three models (KECS, KSE125 and KE125) are selected (in the red dialog box).

According to AIC and C-index, variables were selected step-by-step (Fig. 4), and three models (KECS, KSE125 and KE125) are notable. The KECS model (KPS, EGFR-20, Cy211 and smoking) was the one strictly identified by AIC, the KE125 model (KPS, EGFR-20 and Ca 125) was a simple one with relatively high C-index, and the KSE125 model (KPS, smoking, EGFR-20 and Ca 125) was the one with the highest C-index (77.2%). Additionally, the KTSCS model (KPS, TKI therapy, EGFR-20, Cy211 and smoking) was not evaluated for relatively lower C-index (71.6%).

### Model Evaluation and Validation

The Cox and the 3 RSF models, together with the 2 scoring systems, were separately evaluated in the two groups by C-index, OOB, AIC, and integrated Brier score (Fig. 2).

In the group A, compared with others, the KSE125model had the highest C-index (77.4%), and the lowest OOB and AIC value (25.7% and 725.6). Therefore, the KSE125 model was the best for this cohort. In the 10-fold cross validation of the group A, the patients were randomly divided into 10 parts, and validated the model in each part separately. The performance of the model was evaluated by the integrated Brier score (Fig. 2), which indicated that the KSE125 model (13.2%) was only slightly worse than the Cox model (13.0%) and the KE125 model (13.1%).

In the group B, the 3 RSF models were obviously better than GPA, RPA or Cox model. Although the KECS model’s C-index and OOB (77.4% and 28.6%) were the best, its AIC and IBS were worse than those of the KE125 or KSE125 models in sequence. Above all, compared to others, the KSE125 model developed from the group A performed well in the group B, and the model had both higher prognostic ability and lower overfitting possibility.

Additionally, the results of the group SA and SB (Supplementary Data, Figures S1–S3) confirmed that the KSE125 model had both higher prognostic ability and lower overfitting possibility. Furthermore, compared to other models, the KSE125 model performs obviously better in both groups.

**Figure 5:**
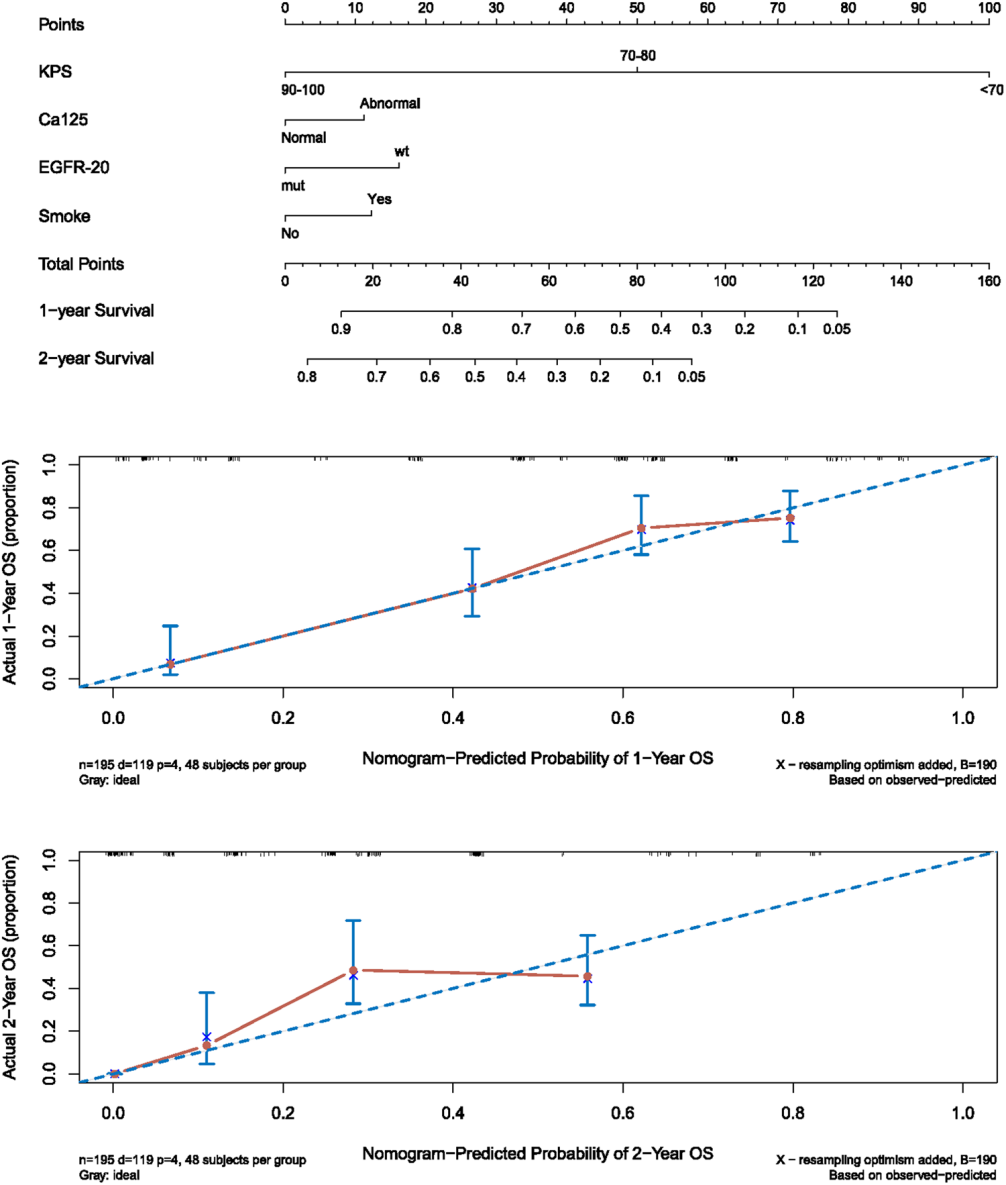
Nomogram (upper) and validation plot (middle and lower) for KSE125 model in all patients. On the nomogram, the 4 predictors for a given patient are projected to the Points axis, and the accumulated total points can be used to predict the 1-year survival rate of the patient.

The prognostic nomogram for the KSE125 model was built for all recruits (Fig. 5A), and its C-index was 75.6% (95% CI: 66.8–84.4%). The predicted 1- year and 2-year OS of the model agreed well with the corresponding actual OS (Fig. 5B and C), and indicated the good performance of the model.

## Discussion

Our study indicates that, for lung adenocarcinoma patients with brain metastasis, a validated prognostic nomogram (KPS, smoking, EGFR-20 and Ca125) can more accurately predict the 1-year and 2-year survival of the patients before TKI therapy than other models.

Many prognostic factors had been related to the survival of NSCLC patients. Besides those in the modified-RPA and the lung-GPA model, the factors also included gene mutations and laboratory indicators^13,24,25^. However, all factors could not be simultaneously included in the prognostic model for overfitting. Therefore, how to use them to develop a model with high predicted ability and low overfitting possibility becomes a problem. In the past, the multivariate Cox regression was popularly applied to select variables. However, as in this cohort, the regression was inferior to RSF in developing prognostic models. Some factors without statistical significance in the Cox model, such as smoking and Ca125, could be integrated into RSF models, and could obviously improve the model’s prognostic ability without apparently increasing overfitting possibility. Above all, as a substitute for the Cox regression method, the RSF based step-by-step variable selection method could be used to develop prognostic models for better meeting the requirement of survival prediction.

Furthermore, our data indicated that the variable selection method could be used to develop reliable models. Using the method, we identified 3 RSF models, which were all confirmed to have both higher prognostic ability and lower overfitting possibility (Fig. 2). Although all of these RSF models could be used to predict the prognosis of the patients, as indicated by the indicators, the KSE125 model was slightly better than others. Additionally, both the models integrated CA125 (KSE125 and KE125) were slightly better than the KECS model, and indicated that CA125 was an important prognostic factor for the patients.

It should note that some patient characteristics were not balanced between the groups (or hospitals). For example, at the diagnosis of lung adenocarcinoma in the group A, more patients already had BM and presented extracranial metastases. And that, less patients in the group A received the combined treatment, which might result in a lower local control rate and shorter median OS. However, the selection bias between the hospitals could not obviously weaken the performance of the KSE125 model in the group B.

In this study, the KSE125 model was superior to others; furthermore, its nomogram performed well in discrimination and calibration. Four variables of the models (KPS, smoking, EGFR-20 and CA125) were all reported as factors for lung adenocarcinoma patients previously^26–29^. Among the factors, KPS which stratified into <70, 70–80 and 90–100 was the key one, and others acted to more accurately correct its prognostic ability. One of such factors was CA-125, which was not in present prognostic models for the patients, but turned out to be a valuable one.

However, this did not mean that all the four factors were the most powerfully independent ones in the prediction. As indicated by minimal depth, although the factors of treatment, already or developed BM and TKI therapy were also powerful, C-index of their combination with other factors was not so high in this cohort of patients (Fig. 4). Above all, the combination of the KSE125 model was better than other variables’.

Regardless of the fact that the KSE125 model was developed from the patients who did not receive TKI therapy before BM, it could also be applied in those who received. According to the study from Sperduto *et al*.^7^, among most patients received TKI therapy before BM, the factor of EGFR was still in the Lung-molGPA model for BM. The prognostic value of EGFR-20 could be explained by that the mutations well responded to TKI therapy^14^, and was classified as a protected predictor on the nomogram (Fig. 4).

Additionally, as indicated by our results and those from Gao *et al*.^30^, some biomarkers from cancer hallmarks have powerful prognostic ability^31^. Currently, more and more these markers were integrated in prediction models; however, overfitting possibility and generalization ability of the models should be thorough evaluated with sufficient sample size. In this study, because only a part of the patients had the information on Alk and Kras mutational status, to develop reliable model, the markers were not evaluated. Considering the importance of the markers in lung adenocarcinoma patients^7^, their prognostic ability would be studied in our future studies.

## Conclusions

For lung adenocarcinoma patients with brain metastasis, a validated prognostic nomogram (KPS, smoking, EGFR-20 and Ca125) can more accurately predict the 1-year and 2-year survival of the patients before TKI therapy than other models.

## Electronic supplementary material

Supplementary Data
