# Supplementary material for: Prognostic value of cancer antigen -125 for lung adenocarcinoma patients with brain metastasis: A random survival forest prognostic model
Source: Sci Rep. 2018 Apr 4;8:5670. doi: 10.1038/s41598-018-23946-7 (PMC5884842; doi:10.1038/s41598-018-23946-7)
Supplement: Supplementary file 1 — Supplementary Data [file 41598_2018_23946_MOESM1_ESM.doc]

**Prognostic value of cancer antigen -125 for lung adenocarcinoma patients with brain metastasis: A random survival forest prognostic model**

Hao Wang^1^†, Liuhai Shen^2^†, Jianhua Geng^3^†, Yitian Wu^3^†, Huan Xiao^2^, Fan Zhang ^1^, Hongwei Si^2*^

**Patient Characteristics**

Patient characteristics of the group SA and SB were well balanced (Table S1), and 35/115 and 9/27 patients developed BM during 0.6-43.1 months (median 5.1 months) and 2.9-23.3 months (median 7.9 months), respectively.

**Survival and Cox Model**

By the end of November 2016, 71/115 patients in the group SA died within 0.6 to 33.0 months (median 8.6 months). In univariate Cox regression, already or developed BM, TKI therapy, EGFR (or EGFR-20), treatment modalities and KPS were significant against OS (Table S2). As the results from the group A, EGFR (OR: 0.468, 95% CI: 0.284-0.772) and KPS (OR: 4.460, 95%CI: 2.885-6.895) were significant predictors identified by the multivariate Cox regression.

**RSF Models**

In the group SA, EGFR-20’s VIMP was higher than EGFR’s (0.0139 vs. 0.0055), and was used to construct RSF models. Minimal depth and VIMP of variables are plotted on Figure S1. The top 13 predictors on the y-axis (Figure S1) were identical to those from the group A; however, the variables excluded by minimal depth (a threshold of 4.1397) and VIMP (zero and negative values) were slightly different. In the group SA, KPS, treatment, TKI therapy, EGFR-20, age, already BM, Ca125 and smoke were qualified for our further analysis.

In the group SA, the step-by-step variable selection by AIC and C-index (Figure S2) indicated that the C-index of KSE125 model was highest among those built by non-treatment related factors. Furthermore, the model having the highest C-index on Figure S2 is KSE125 augmented with treatment related predictors (treatment modalities and TKI therapy). Above all, using data of the group SA could develop the KSE125 model which had both higher prognostic ability and lower overfitting possibility.

**Model Evaluation and Validation**

As in the group A, C-index, OOB, integrated Brier score, and AIC were used to evaluate the performance of KSE125 model, Cox model, lung-GPA and modified RPA. Figure S3 indicated that, compared to other models, the KSE125 model performs obviously better in both group SA and SB.

Table S1: Patient characteristics of group SA and SB.

Table S2: Univariate Cox regression for the group SA.

Figure S1: Scatter plot of VIMP against minimal depth.

Figure S2: Variable selected by AIC and C-index.

Figure S3: Comparison of prognostic models for lung adenocarcinoma patients with brain metastasis in the group SA and SB

**Tables**

Table S1. Patient characteristics of group SA and SB.

| Characteristic |  | n (%) | Group SA (n=115) | Group SB (n=27) | *x*^2^ |
| --- | --- | --- | --- | --- | --- |
| Gender | Male | 70 (49.3) | 56(48.7) | 14(51.9) | 0.087 |
|  | Female | 72 (50.7) | 59(51.3) | 13(48.1) |  |
| Age (year) | <50 | 36 (25.4) | 30(26.1) | 6(16.7) | 2.844 |
|  | 50-59 | 49 (34.5) | 36(31.3) | 13(48.1) |  |
|  | ≥60 | 57 (40.1) | 49(42.6) | 8(29.6) |  |
| Smoking | No | 87 (61.3) | 70(60.9) | 17(63.0) | 0.040 |
|  | Yes | 55 (38.7) | 45(39.1) | 10(37.0) |  |
| CEA | Normal | 68 (47.9) | 54(47.0) | 14(51.9) | 0.210 |
|  | Abnormal | 74 (52.1) | 61(53.0) | 13(48.1) |  |
| CA199 | Normal | 103 (72.5) | 85(73.9) | 18(66.7) | 0.576 |
|  | Abnormal | 39 (27.5) | 30(26.1) | 9(33.3) |  |
| SCC | Normal | 137 (96.5) | 112(97.4) | 25(92.6) | 1.482 |
|  | Abnormal | 5 (3.5) | 3(2.6) | 2(7.4) |  |
| NSE | Normal | 135 (95.1) | 108(93.9) | 27(100) | 1.729 |
|  | Abnormal | 7 (4.9) | 7(6.1) | 0 |  |
| Primary | Controlled | 19 (13.4) | 15(13.0) | 4(14.8) | 0.059 |
| tumor | Uncontrolled | 123 (86.6) | 100(87.0) | 23(85.2) |  |
| Extracranial | Yes | 110 (77.5) | 88(76.5) | 22(81.5) | 0.308 |
| metastases | No | 32 (22.5) | 27(23.5) | 5(18.5) |  |
| NoBM | 1 | 51 (35.9) | 41(35.7) | 10(37.0) | 0.144 |
|  | 2-3 | 41 (28.9) | 34(29.6) | 7(25.9) |  |
|  | >3 | 50 (35.2) | 40(34.8) | 10(37.0) |  |
| ProGRP | Normal | 141 (99.3) | 114(99.1) | 27(100) | 0.236 |
|  | Abnormal | 1 (0.7) | 1(0.9) | 0 |  |
| Treatment | No treatment | 17 (12.0) | 14(12.2) | 3(11.1) | 7.083 |
|  | Chemotherapy | 42 (29.6) | 33(28.7) | 9(33.3) |  |
|  | Radiotherapy | 28 (19.7) | 22(19.1) | 6(22.2) |  |
|  | Combined | 39 (27.5) | 36(31.3) | 3(11.1) |  |
|  | TKI alone | 16 (11.3) | 10(8.7) | 6(22.2) |  |
| EGFR | wt | 73 (51.4) | 60(52.2) | 13(48.1) | 0.142 |
|  | mut | 69 (48.6) | 55(47.8) | 14(51.9) |  |
| EGFR-20 | wt | 76 (53.5) | 63(54.8) | 13(48.1) | 0.387 |
|  | mut | 66 (46.5) | 52(45.2) | 14(51.9) |  |
| Cy211 | Normal | 45 (31.7) | 37(32.2) | 8(29.6) | 0.065 |
|  | Abnormal | 97 (68.3) | 78(67.8) | 19(70.4) |  |
| CA125 | Normal | 74 (52.1) | 61(53.0) | 13(48.1) | 0.210 |
|  | Abnormal | 68 (47.9) | 54(47.0) | 14(51.9) |  |
| TKI therapy | No | 83 (58.5) | 68(59.1) | 15(55.6) | 0.247 |
|  | in wt pt | 12 (8.5) | 10(8.7) | 2(7.4) |  |
|  | In mut pt | 47 (33.1) | 37(32.2) | 10(37.0) |  |
| BM | Already | 98 (69.0) | 80(69.6) | 18(66.7) | 0.086 |
|  | Developed | 44 (31.0) | 35(30.4) | 9(33.3) |  |
| KPS | <70 | 40 (28.2) | 35(30.4) | 5(18.5) | 1.680 |
|  | 70-80 | 86 (60.6) | 68(59.1) | 18(66.7) |  |
|  | 90-100 | 16 (11.3) | 12(10.4) | 4(14.8) |  |
| modified-RPA | Classes I | 1 (0.7) | 0 | 1(3.7) | 5.569 |
|  | Classes II | 101 (71.1) | 80(69.6) | 21(77.8) |  |
|  | Classes III | 40 (28.2) | 35(30.4) | 5(18.5) |  |
| lung-GPA | 0-1 | 52 (36.6) | 42(36.5) | 10(37.0) | 0.189 |
|  | 1.5-2.5 | 77 (54.2) | 63(54.8) | 14(51.9) |  |
|  | 3 | 9 (6.3) | 7(6.1) | 2(7.4) |  |
|  | >3.5 | 4 (2.8) | 3(2.6) | 1(3.7) |  |

Table S2. Univariate Cox regression for the group SA.

| Characteristic |  | n | 1-year OS (%) | Median (moths) | p |
| --- | --- | --- | --- | --- | --- |
| Gender | Male | 56 | 44.4 | 9.6 | 0.292 |
|  | Female | 59 | 50.1 | 13.9 |  |
| Age (year) | <50 | 30 | 46.6 | 11.4 | 0.921 |
|  | 50-59 | 36 | 50.2 | 13.2 |  |
|  | ≥60 | 49 | 45.7 | 10.2 |  |
| Smoking | No | 70 | 49.5 | 11.8 | 0.273 |
|  | Yes | 45 | 43.6 | 8.6 |  |
| CEA | Normal | 54 | 47.0 | 10.2 | 0.961 |
|  | Abnormal | 61 | 47.5 | 9.6 |  |
| CA199 | Normal | 85 | 46.2 | 10.2 | 0.374 |
|  | Abnormal | 30 | 49.4 | 8.6 |  |
| SCC | Normal | 112 | 48.1 | 11.4 | 0.644 |
|  | Abnormal | 3 | 0 | 9.7 |  |
| NSE | Normal | 108 | 47.5 | 10.2 | 0.686 |
|  | Abnormal | 7 | 42.9 | 6.9 |  |
| Primary tumor | Controlled | 15 | 58.7 | 13.2 | 0.951 |
|  | Uncontrolled | 100 | 45.8 | 9.7 |  |
| Extracranial | Yes | 88 | 46.8 | 9.7 | 0.929 |
| metastases | No | 27 | 49.2 | 11.4 |  |
| NoBM | 1 | 41 | 54.7 | 12.5 | 0.537 |
|  | 2-3 | 34 | 40.2 | 9.7 |  |
|  | >3 | 40 | 45.6 | 8.5 |  |
| ProGRP | Normal | 114 | 47.8 | 10.2 | 0.396 |
|  | Abnormal | 1 | 0.0 | 6.9 |  |
| Cy211 | Normal | 37 | 54.5 | 12.5 | 0.356 |
|  | Abnormal | 78 | 43.8 | 8.9 |  |
| CA125 | Normal | 61 | 52.8 | 14.3 | 0.163 |
|  | Abnormal | 54 | 40.8 | 8.9 |  |
| Treatment | No treatment | 14 | 14.3 | 3.1 | 0.003 |
|  | Chemotherapy | 33 | 47.1 | 11.8 |  |
|  | Radiotherapy | 22 | 58.0 | 14.8 |  |
|  | Combined | 36 | 46.7 | 11.4 |  |
|  | TKI alone | 10 | 80.0 | NA |  |
| EGFR | wt | 60 | 35.9 | 7.0 | 0.001 |
|  | mut | 55 | 60.4 | 16.4 |  |
| EGFR-20 | wt | 63 | 35.8 | 7.0 | 0.000 |
|  | mut | 52 | 61.9 | 21.9 |  |
| TKI therapy | No | 68 | 32.4 | 6.9 | 0.000 |
|  | in wt pt | 10 | 70.0 | 15.2 |  |
|  | In mut pt | 37 | 69.8 | NA |  |
| BM | Already | 80 | 51.5 | 12.5 | 0.008 |
|  | Developed | 35 | 37.9 | 6.1 |  |
| KPS | <70 | 35 | 9.3 | 3.2 | 0.000 |
|  | 70-80 | 68 | 56.9 | 13.9 |  |
|  | 90-100 | 12 | 83.3 | NA |  |
| modified-RPA | Classes I | 0 | NA | NA | 0.000 |
|  | Classes II | 80 | 62.3 | 15.2 |  |
|  | Classes III | 35 | 5.9 | 3.2 |  |
| lung-GPA | 0-1 | 42 | 32.7 | 6.1 | 0.008 |
|  | 1.5-2.5 | 63 | 51.9 | 12.5 |  |
|  | 3 | 7 | 71.4 | 16.4 |  |
|  | >3.5 | 3 | 100.0 | 19.6 |  |

**Figures**


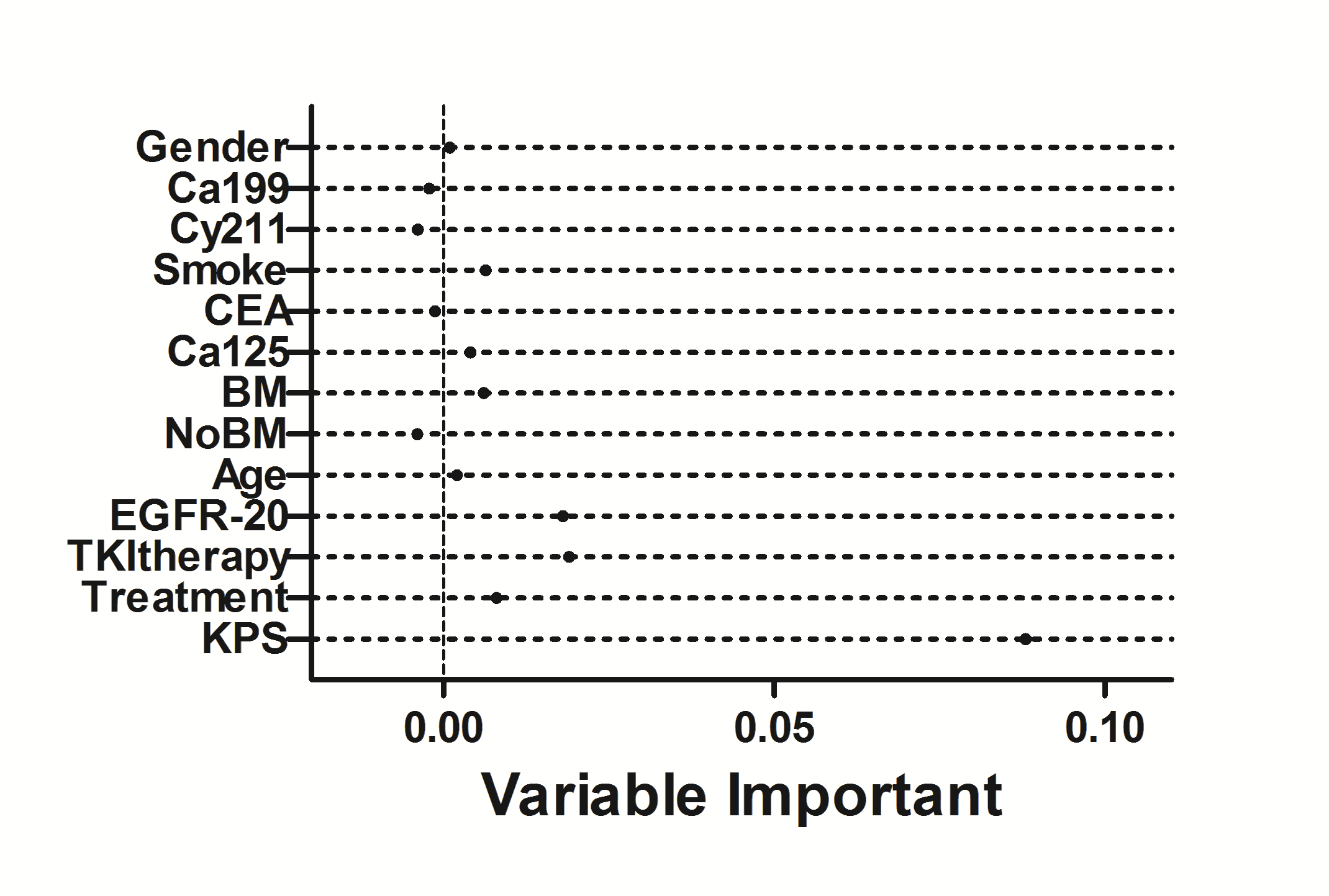


Figure S1: Scatter plot of VIMP against minimal depth. The variables on y-axis are sorted by minimal depth, and the smaller is closer to the origin. Additionally, only variables with a minimal depth lower than the threshold (4.1397) are plotted.


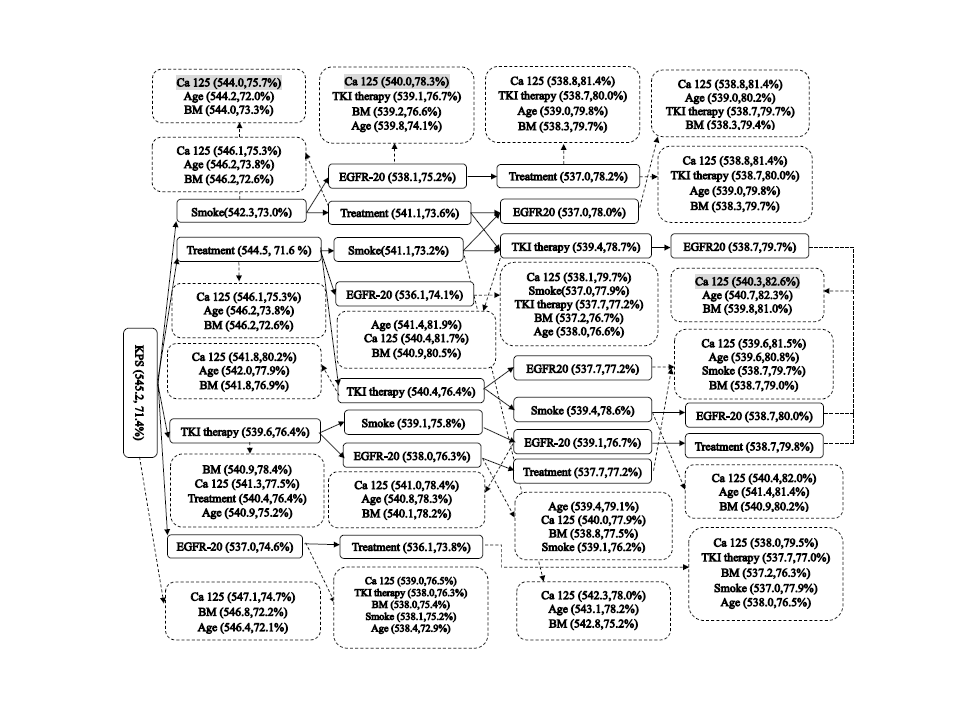


Figure S2: Variable selected by AIC and C-index. Broken lines indicate the ruled out variables. The last variables of eligible models are masked in grey background.


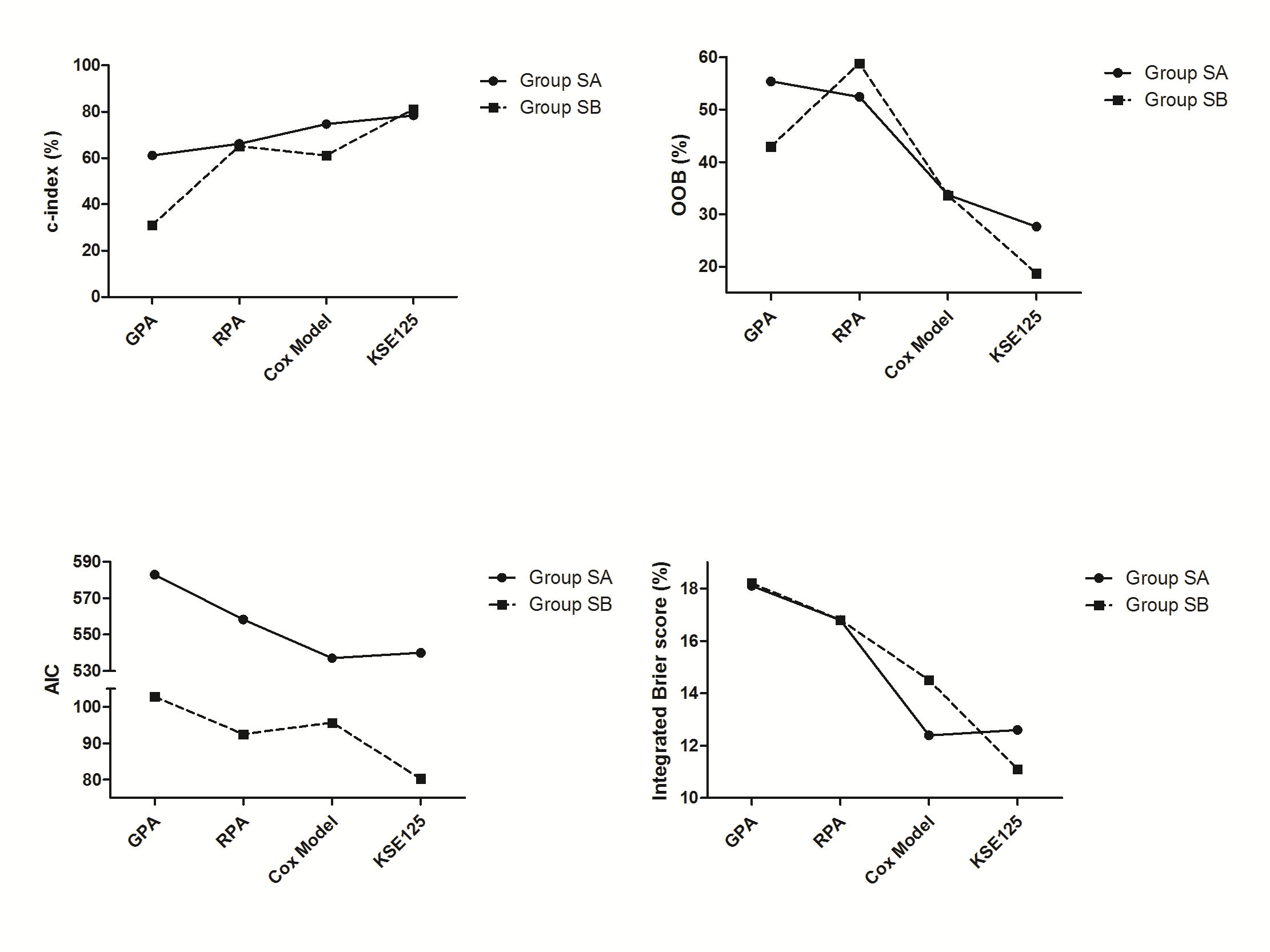


Figure S3: Comparison of prognostic models for lung adenocarcinoma patients with brain metastasis in the group SA and SB
